# Supplementary material for: Novel Driver Strength Index highlights important cancer genes in TCGA PanCanAtlas patients
Source: PeerJ. 2022 Aug 11;10:e13860. doi: 10.7717/peerj.13860 (PMC9375969; doi:10.7717/peerj.13860)
Supplement: Supplemental Information 2 [file peerj-10-13860-s002.zip › COHORTS/genes plots/age/2021_8_16_14_9_distribution_cohorts_60-64.pdf]

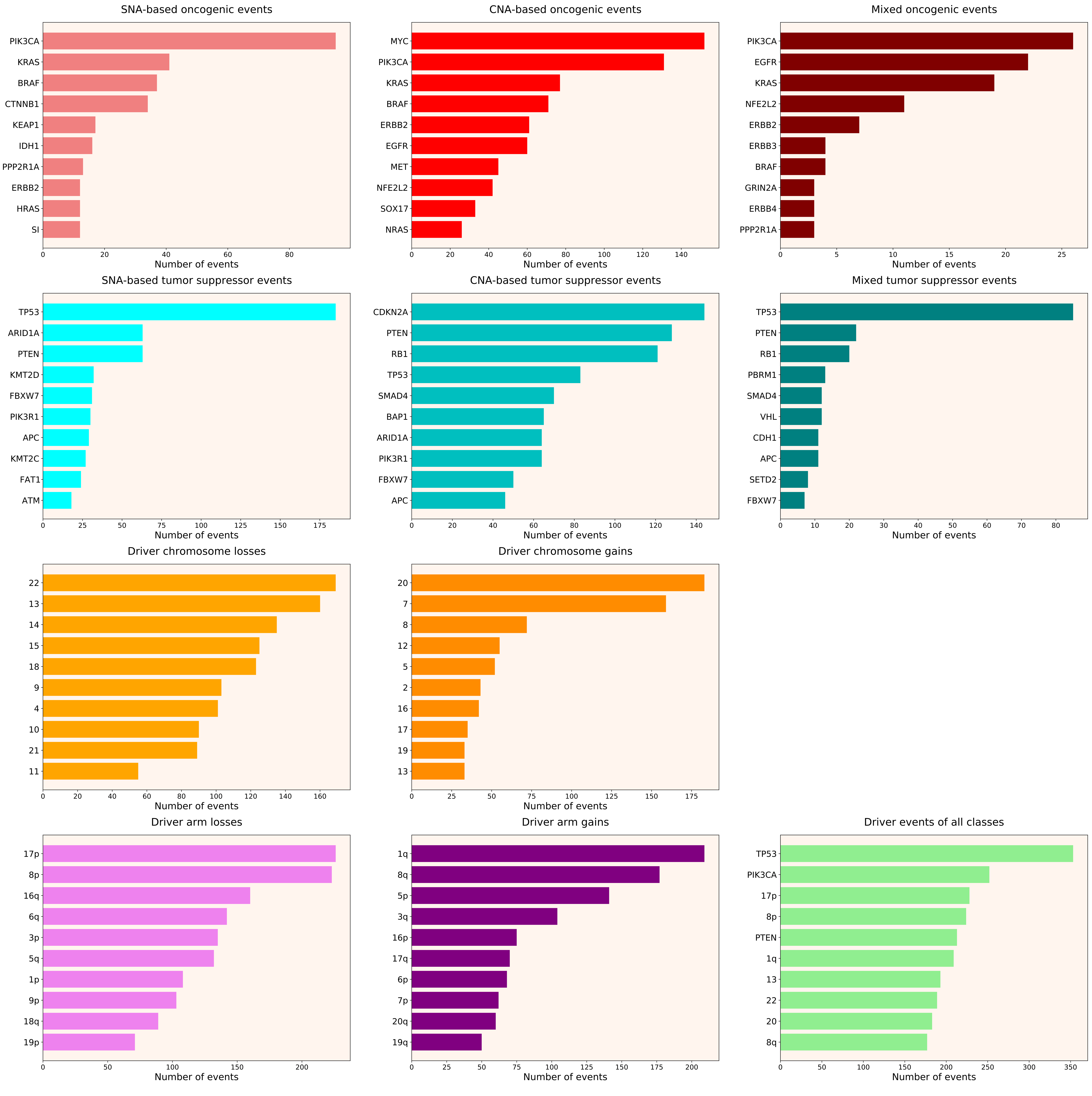
SNA-based oncogenic events
PIK3CA
KRAS
BRAF
CTNNB1
KEAP1
IDH1
PPP2R1A
ERBB2
HRAS
SI
0 20 40 60 80
Number of events
CNA-based oncogenic events
MYC
PIK3CA
KRAS
BRAF
ERBB2
EGFR
MET
NFE2L2
SOX17
NRAS
0 20 40 60 80 100 120 140
Number of events
Mixed oncogenic events
PIK3CA
EGFR
KRAS
NFE2L2
ERBB2
ERBB3
BRAF
GRIN2A
ERBB4
PPP2R1A
0 5 10 15 20 25
Number of events
SNA-based tumor suppressor events
TP53
ARID1A
PTEN
KMT2D
FBXW7
PIK3R1
APC
KMT2C
FAT1
ATM
0 25 50 75 100 125 150 175
Number of events
CNA-based tumor suppressor events
CDKN2A
PTEN
RB1
TP53
SMAD4
BAP1
ARID1A
PIK3R1
FBXW7
APC
0 20 40 60 80 100 120 140
Number of events
Mixed tumor suppressor events
TP53
PTEN
RB1
PBRM1
SMAD4
VHL
CDH1
APC
SETD2
FBXW7
0 10 20 30 40 50 60 70 80
Number of events
Driver chromosome losses
22
13
14
15
18
9
4
10
21
11
0 20 40 60 80 100 120 140 160
Number of events
Driver chromosome gains
20
7
8
12
5
2
16
17
19
13
0 25 50 75 100 125 150 175
Number of events
Driver arm losses
17p
8p
16q
6q
3p
5q
1p
9p
18q
19p
0 50 100 150 200
Number of events
Driver arm gains
1q
8q
5p
3q
16p
17q
6p
7p
20q
19q
0 25 50 75 100 125 150 175 200
Number of events
Driver events of all classes
TP53
PIK3CA
17p
8p
PTEN
1q
13
22
20
8q
0 50 100 150 200 250 300 350
Number of events
